# Translation and psychometric assessment of a Persian version of medication safety competence scale (MSCS) for clinical nurses

**DOI:** 10.1038/s41598-023-29399-x

**Published:** 2023-02-08

**Authors:** Fateme Mohammadi, Seyed Amin Kouhpayeh, Mostafa Bijani, Mojtaba Farjam, Amir Faghihi, Zohreh Badiyepeymaiejahromi

**Affiliations:** 1 Chronic Diseases (Home Care) Research Center and Autism Spectrum Disorders Research Center, Department of Nursing, Hamadan University of Medical Sciences, Hamadan, Iran; 2 Department of Pharmacology, School of Medicine, Fasa University of Medical Sciences, Fasa, Iran; 3 Department of Medical Surgical Nursing, School of Nursing, Fasa University of Medical Sciences, Fasa, Iran; 4 Department of Pharmacology, School of Medicine. Noncommunicable Diseases Research Center (NCDRC), Fasa University of Medical Sciences, Fasa, Iran; 5 Faculty of Nursing, Jahrom University of Medical Sciences, Jahrom, Iran

**Keywords:** Health care, Health occupations, Medical research

## Abstract

Nurses play a key role in medication safety and, by extension, patient safety. Evaluation of medication safety competence in nurses requires valid, specific, and comprehensive instruments. The present study was conducted to translate and psychometric assessment a Persian version of medication safety competence scale (MSCS) for clinical nurses in Iran. This is a cross-sectional and multi-centric work of research with a methodological design. A total of 1080 clinical nurses were selected from 5 cities located in Iran. The original version of the MSCS was translated into Persian and the psychometric properties of MSCS were assessed using COSMIN criteria. The exploratory factor analysis (EFA) showed that the factor loading of the 36 items was between 0.72–0.87, all of which were significant. The confirmatory factor analysis (CFA) fitted the data well (χ^2^/df = 7, RMSEA = 0.01, CFI = 0.96, NFI = 0.95, and TLI = 0.97). The reliability of the instrument was assessed in terms of its internal homogeneity where the Cronbach's alpha of the whole instrument was found to be 0.96. The Persian version of MSCS for nurses possesses satisfactory validity and reliability. Thus, nurse managers can use this instrument to measure medication safety competence in nurses.

## Introduction

As one of the key indexes of the quality of healthcare services, patient safety is defined as protecting patients from any harm or injury while care is being provided to them^1^, and one of the key factors in patient safety is medication safety^2^. According to a report released by Federal University of Sao Paulo about medication safety, medication errors or the side effects of medication account for 7% of hospitalization cases in the healthcare system and, every year, 44,000 to 98,000 deaths occur as a result of medication errors in the U.S., which impose 17 to 29 billion dollars on the healthcare system^3^. According to a recent meta-analysis, the prevalence of medication errors is between 7.1 and 90%^4^. Another study reports the prevalence of medication errors made by nurses to be between 16 and 44.4%^5^. In a study conducted in Iran, 66.7% of the nurses in special care units had committed medication errors and 40.6% of the nurses reported at least one medication error^6^. In 2017, the World Health Organization described the increase in fatalities due to medication administration errors as a major threat to the safety and health of patients and urged healthcare policy makers worldwide to make a global commitment to decrease the occurrence of medication errors and the ensuing consequences by employing effective preventive strategies^7^.

Medication safety has become a main concern of healthcare systems all over the world^8^. Medication safety means protection against accidental harms and avoidance of any preventable side effects while using medication, achievement of maximum therapeutic effect, and occurrence of minimum side effects^9^. The goal of safe medication therapy is to put patients at the center of attention, provide them with comprehensive medicinal services, and ensure the appropriateness of medications and improvement in patients’ health^10^. One of the main responsibilities of nurses is safe administration of medication; therefore, in view of the high prevalence of medication errors, it is important to consider the role of nurses’ medication safety competence in patient safety^11^. Yet, only a few studies have addressed the significance of medication safety competence in clinical nurses’ performance and there is a lack of instruments specifically designed to measure this type of competence^12,13^.

In July 2020, researchers in Korea developed and evaluated the Medication Safety Competence Scale (MSCS) for nurses^14^. MSCS measures nurses’ competence in terms of their knowledge, skills, and attitude in relation to medication safety and can help improve the quality of clinical care, research and education. Moreover, as a scale supported by a 6-factor structure, MSCS can provide a thorough evaluation of medication safety competence from different aspects, which makes it superior to a single-dimension scale. The Medication Safety Competence Scale developed and evaluated by Park and Seomun in Korea consists of 36 items and addresses six domains: medication management and patient assessment, improvement of safety problems in the medication process, management of effecting factors, safety risk management, multidisciplinary collaboration, and responsibility as a professional nurse^14^. In Iran, there is lack of a standard instrument which specifically evaluates nurses’ medication safety competence^15–17^.

As a result, medication safety competence is not properly measured and managers rely on the reports of medication errors cited by nurses or head nurses. This subject prevents the strengths and weaknesses of nurses in the field of medication safety from being accurately identified, leading to an increase in medication errors. In their evaluation of clinical performance, nurse managers and clinical trainers attach great importance to safe nursing and patient safety. However, medication safety competence in nurses is not measured effectively, because there is not a standard and comprehensive instrument.

In Iran, there is lack of a standard instrument which specifically evaluates nurses’ medication safety competence. As a result, medication safety competence is not properly measured and managers rely on the reports of medication errors cited by nurses or head nurses, which prevents the strengths and weaknesses of nurses in the field of medication safety from being accurately identified, leading to an increase in medication errors. In their evaluation of clinical performance, nurse managers and clinical trainers attach great importance to safe nursing and patient safety; however, medication safety competence in nurses is not measured effectively because there is not a standard and comprehensive instrument which specifically addresses nurses’ competence in this area.

In view of the great importance of medication safety and the fact that assessment of medication safety competence in nurses requires a valid and specific instrument which is lacking in Iran. The present study aims to translate and psychometric assessment a Persian version of medication safety competence scale for clinical nurses in Iran.

## Methods

### Study design and settings

The present methodological study was conducted from November 2021 to February 2022. The study context was five hospitals located in Fars provinces of Iran. There are specialized and super-specialized services in the departments of Surgery, Emergency, Internal medicine, ICU, CCU, Dialysis and Pediatrics in five hospitals. The psychometric properties of the MSCS, including content validity, reliability (internal consistency and stability) and construct validity (exploratory factor analysis and confirmatory factor analysis), were evaluated. The psychometric properties of the MSCS were assessed using COSMIN (Consensus-based Standards for the selection of health Measurement Instruments) criteria^18^. Also, the COSMIN checklist was used to evaluate the methodological quality of studies conducted on the measurement properties of the scale^19^.

### Sample size, inclusion and exclusion criteria

The sample size for evaluation of the psychometric properties of MSCS was calculated based on the number of inventory sections, resulting in 10 subjects per item^20^. However, in this study, about 30 respondents per item were recruited to ensure the accuracy of exploratory factor analysis and confirmatory factor analysis. Participants were selected by convenience sampling from five hospitals located in Iran. 1080 nurses participated in exploratory factor analysis and 1080 nurses participated in confirmatory factor analysis. The nurses participating in exploratory factor analysis were other than the nurses participating in confirmatory factor analysis.

The inclusion criteria were (1) Being willing to participate in the study, (2) Being a nursing school graduate, (3) Being in practice in a hospital department or clinic, (4) Having at least one year’s work experience, (5) Not having a history of psychological or emotional disorder (depression and anxiety disorder, bipolar disorder. etc.). (6) All the participants gave written informed consent to participate in the study. The subjects, who failed to answer over half of the questions on the questionnaires, did not return the questionnaires were excluded.

### The medication safety competence scale (MSCS)

The Medication Safety Competence Scale (MSCS) is a self-report questionnaire based on a five-point Likert scale scoring system (1–5). MSCS consists of 36 items classified into six dimensions. The scale has been designed to be completed within 20 min. The total score range is from 36 to 180. Higher scores indicate better medication safety competence^14^.

### Translation procedures and cultural adaptation

Before being translated, the questionnaire was procured and permission was obtained from the developers. The questionnaire was then translated according to the translation and cross-cultural adaptation guideline from Beaton et al.^21^. Accordingly, the English version of the MSCS was first translated into Persian with forward- backward approach in six steps; (1) In the forward translation stage, two bilingual translators, who were native speakers of English and Persian and also familiar with Iranian culture, translated the English text into Persian. (2) Then two translations were reviewed by two translators and the research team and synthesized into a single translation. (3) In the back translation stage, the Persian questionnaire prepared from the previous stage was given to a bilingual translator fluent in Persian and English and asked to translate the questionnaire from Persian to English. (4) In the Expert Committee stage, a committee of instrumentation specialists, nurses, doctors and translators was formed and reviewed the translation versions of the questionnaire from the previous stages and reached a consensus on a single version. (5) In test of the pre final version, the 4th stage version was evaluated. Therefore, 25 nurses were randomly selected and asked to assess the revised Persian scale. Based on their feedback, the scale was revised and improved. (6) In submission of scale finally, the psychometric properties of MSCS, including its content validity, reliability (internal consistency and stability) and construct validity, were evaluated and reported.

### Statistical analysis [psychometric properties (COSMIN criteria)]

#### Face validity and content validity

The revised questionnaire was given to 30 practicing nurses who were asked to assess each item in terms of relevance, appropriate use of grammar and vocabulary, and intelligibility. They evaluated each item using a 5-point Likert scale ranging from 1 (not important at all) to 5 (very important). Finally, all the questionnaires were collected and analyzed. Impact scores of greater than 1.5 were considered acceptable^22^. Thirty experts were chosen based on the following inclusion criteria: having a minimum of a bachelor’s degree in nursing and at least one year’s experience of professional practice in clinical settings. MSCS was given to 15 nurses with a PhD in nursing and 15 practicing nurses from 5 hospitals. These professionals evaluated each item in terms of use of vocabulary and grammar, intelligibility, and relevance to the Iranian culture. They also provided comments next to each item. The questionnaires were returned to the experts to assess the content validity ratio (CVR). They were asked to assess the items in terms of usefulness and necessity. Next, the content validity of each item was measured. The revised version of MSCS was resubmitted to the 30 participants who were asked to give each item a score in terms of relevance, simplicity and clarity on a four-point Likert scale ranging from 1 to 4. The content validity index (CVI) was calculated for each item and MSCS as a whole. In this study, CVI > 0.8 and CVR > 0.33 were considered appropriate^23^.

#### Construct validity (exploratory factor analysis and confirmatory factor analysis)

Evaluation of construct validity was executed to ensure that the instrument actually measured what it was designed to measure^23^. In this stage, exploratory factor analysis was implemented, based on the maximum likelihood method of extracting and varimax rotation^24^. To achieve optimal structure, the researchers used the following criteria: eigenvalues of higher than 1.0, and factor loadings of higher than 0.50^20^. The adequacy of the samples was evaluated using the Kaiser–Meyer–Olkin (KMO) test for sampling adequacy and Bartlett’s test before exploratory factor analysis. For exploratory factor analysis, the KMO value had to be greater than 0.7, and Bartlett’s test value had to be less than 0.05 (P < 0.05).

If the factor loading for each item is less than 0.5, it will be removed from the questionnaire. For evaluation of construct validity, the desirable sample size was estimated to be 10 times the number of items in the inventory^25^. In this study, 30 nurses were considered for each item, and, thus, 1080 nurses participated in the evaluation of construct validity stage. But the factor loading of all items was greater than 0.5 and no item was deleted. Confirmatory factor analysis was executed with 1080 practicing nurses other than the participants in the exploratory factor analysis. Confirmatory factor analysis was conducted using AMOS (v. 21.0) and several indices were employed to measure the usefulness of the model. The following requirements needed to be met: goodness of fit index (GFI) greater than 0.90, root mean square error of approximation (RMSEA) with acceptance level of smaller than 0.08, Tucker Lewis Index (TLI) with acceptance level of greater than 0.90, and comparative fit index (CFI) with acceptance level of greater than 0.90^26^.

#### Reliability (internal consistency and stability)

The reliability of this instrument was measured using the Cronbach’s alpha coefficient with type construct reliability and test–retest reliability. For evaluation of internal consistency, the Cronbach’s alpha coefficient was calculated for 1080 samples; a Cronbach’s alpha coefficient of greater than 0.7 was considered to be acceptable^27^. As for test–retest reliability, the intra class correlation (ICC) of the scale was calculated by collecting data from 300 practicing nurses with a two-week interval. If the ICC index of an instrument is above 0.80, its consistency is considered as satisfactory^28^.

### Ethical considerations

All the participants gave written informed consent to participate in the study. The present study was conducted in terms of the principles of the revised Declaration of Helsinki, which is a statement of ethical principles that directs physicians and other participants in medical research involving human subjects. The participants were assured about their anonymity and confidentiality of their information. Moreover, the study was approved by the Institutional Research Ethics Committee of Fasa University of Medical Sciences, Fasa, Iran (Ethical code: IR.FUMS.REC.1400.092). All methods were performed in accordance with the relevant guidelines and regulations, and all the research methods met the ethical guidelines described in the Declaration of Helsinki.

## Results

### The socio-demographic characteristics of the nurses

The ages of the practicing nurses who participated in the study ranged between 23 and 53 years, with the mean being 33.30 ± 7.09 years. The mean of their work experience was 9.22 ± 6.65 years. (Table 1) shows the participants’ demographic characteristics.

**Table 1:** Frequency distribution of demographic characteristics (n = 1080).

Variable	N	%
Gender		
Male	278	25.74
Female	802	74.26
Marital status		
Unmarried	300	27.77
Married	702	65
Divorced/widowed	78	7.23
Education level		
Bachelor's degree in nursing	892	82.59
Master degree in nursing	143	13.25
PhD degree in nursing	45	4.16
Work experience (year)		
1–5	304	28.14
6–10	223	20.64
11–15	328	30.37
> 15	225	20.85
Ward		
Surgical	131	12.12
Internal	302	27.96
I.C.U	92	8.51
C.C.U	102	9.44
Emergency	261	24.16
Infectious	70	6.50
Pediatric	60	5.55
Dialysis	30	2.30
General neurology	32	2.96

#### Face validity

In this stage of the study, the participating nurses reported that all the rated items were simple, clear, and related to the subject of the study. Moreover, the impact score was higher than 1.5 for all the items. Any items were not removed from this scale during present study.

#### Content validity

In qualitative content analysis, 30 of the nurses suggested that three items (4, 23, and 31) in the Persian script should be rewritten for better clarity and understanding of meaning and concept. After being rewritten, these three items were re-examined and approved by the experts. Based on the experts’ views on the necessity of the items, the CVR was calculated. According to the Lawshe table, the acceptable value of CVR is 0.33. The CVR of all the items of MSCS ranged from 0.46 to 1; therefore, no items were removed because of unsatisfactory CVR. The CVI of each item was also calculated and found to range from 0.80 to 1. None of the items had a score below this cut-off point and all the items were retained. Finally, the SCVI/Average of MSCS was found to equal 0.96.

#### Construct validity

**Figure 1:**
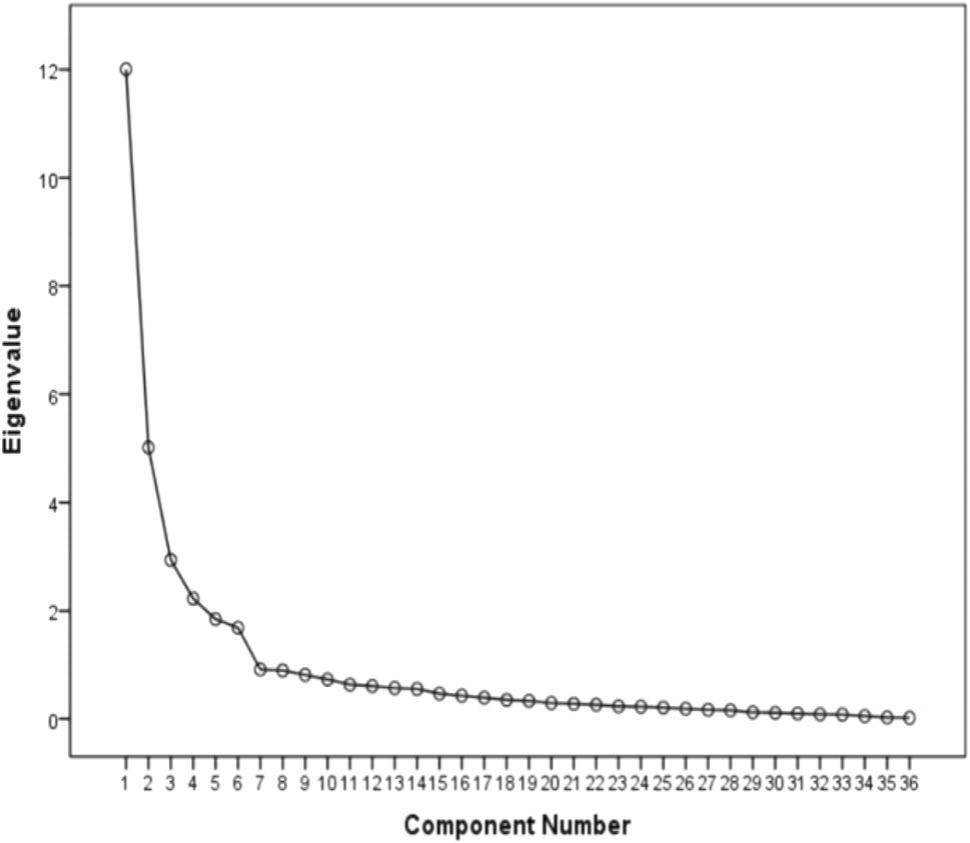
Scree plot of exploratory factor analysis for Persian version of the medication safety competence scale.

The first step in exploratory factor analysis is calculating KMO (Fig. 1). The KMO of the present scale was 0.90, demonstrating the adequacy of the sample for analysis. Also, the factor loading of all items was greater than 0.5 and no item was deleted. The factor analysis results showed that six factors had special values greater than 1 and explained 84.93% of the total variance (χ^2^ = 43,756.314; P < 0.001). Based on the Scree plot, six factors were confirmed for the questionnaire (Fig. 1). The findings also showed that the items’ factor loadings ranged from 0.72 to 0.87. The factor loadings are shown in (Table 2).

**Table 2:** Varimax factor loadings of the items of the instrument (n = 1080).

Factors’ names	Item	Communality	Factor loading
Factor 1: patient-centered medication management	1. Planning care in the medication process	0.81	0.87
	2. Communicating individually according to patients’ condition and level in the medication process	0.072	0.81
	3. Evaluating my nursing practice in the medication process	0.65	0.78
	4. Giving confidence to patients and caregivers in the medication process	0.74	0.80
	5. Giving a sense of stability through clear and consistent communication with patient	0.69	0.81
	6. Documentation of assessment, planning, administration of medication, and evaluation of outcomes	0.72	0.78
	7. Effective patient training to help patients speak of the symptoms of adverse effects	0.79	0.85
	8. Practicing medication care with responsibility for the safety of patients	0.73	0.86
	9. Detecting adverse reactions in medication	0.71	0.82
Factor 2: improvement of safety problems	10. Improving the complex and vulnerable way of medication safety(e.g., incorrect administration practices)	0.68	0.81
	11. Establish prevention measures when medication errors or near-misses occur	0.74	0.84
	12. Trying to create a supportive environment that encourages people to talk about problems when medication errors	0.70	0.80
	13. Identifying the root cause rather than blaming the individual when medication errors or near-misses occur	0.69	0.73
	14. Establishing prevention measures when adverse drug events occur	0.75	0.84
	15. Having a questioning attitude and speaking up when you see something that may be unsafe	0.71	0.81
	16. Analyzing the case to find the root cause of the medication error	0.63	0.72
	17. Reporting to a nursing manager or supervisor when medication errors or near-misses occur	0.69	0.81
Factor 3: management of effecting factors	18. Understanding the role of environmental factors such as workflow and resources, which effect medication safety	0.71	0.80
	19. Understanding the role of human factors, such as fatigue, that affect medication safety	0.72	0.85
	20. Finding information about medication from different sources	0.68	0.81
	21. Describing prevention activities for medication safety	0.61	0.84
	22. Administration according to the right way (patient, drug, dose, route, and time)	0.72	0.86
	23. Using information technology and computerized systems for medication safety	0.66	0.75
Factor 4: safety risk management	24. Coping quickly according to hospital protocol when adverse drug events occur	0.64	0.81
	25. Coping quickly according to hospital protocol when medication errors or near-misses occur	0.61	0.73
	26. Reporting the adverse drug events according to the reporting system	0.71	0.84
	27. Reporting the medication errors or near-misses according to the reporting system	0.68	0.81
	28. Assess the need for medication by checking patients’ condition and examination results prior to administration	0.62	0.72
	29. Managing the medicine according to the hospital’ s medication management guidelines	0.72	0.83
Factor 5: multidisciplinary collaboration	30.Collaborating with multidisciplinary professionals to address medication safety issues	0.69	0.84
	31. Communicating effectively between multidisciplinary members to address medication safety issues	0.65	0.82
	32. Sharing decision-making between multidisciplinary to address medication safety issues	0.71	0.85
	33. Collaborating with other departments for medication safety	0.67	0.81
Factor 6: responsibility in the nursing profession	34. Receiving regularly medication safety training	0.70	0.84
	35. Evaluating regularly my knowledge of medication safety	0.62	0.79
	36. Performing medication care with the alertness as the professional	0.71	0.82

#### Confirmatory factor analysis

**Figure 2:**
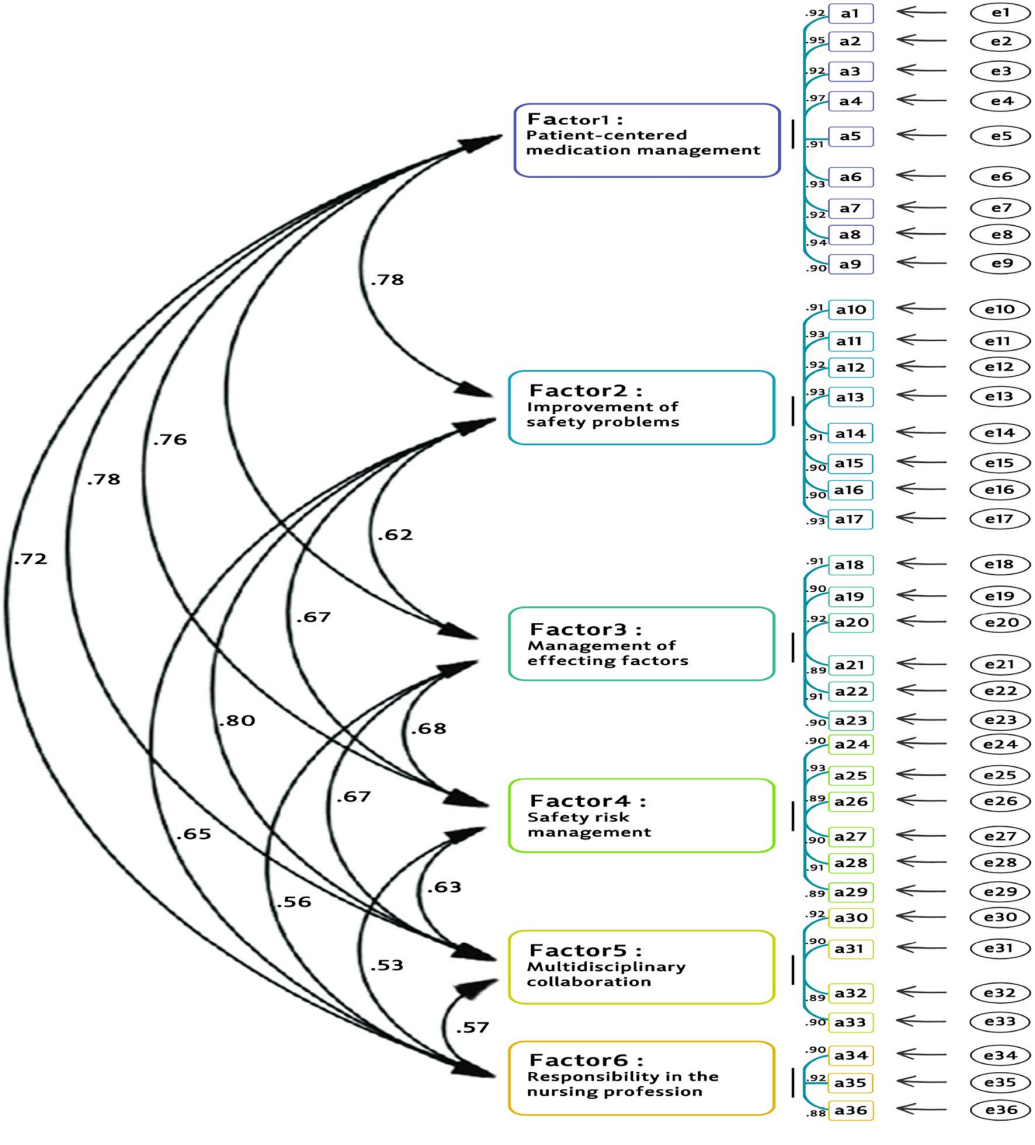
Confirmatory factor analysis model of the medication safety competence scale (N = 1080).

The results of confirmatory factor analysis showed one model with 6 factors. The factors consisted of patient-centered medication management (9 items), improvement of safety problems (8 items), management of effecting factors (6 items), safety risk management (6 items), multidisciplinary collaboration (4 items), and responsibility in the nursing profession (3 items). The correlation of factors 1 to 6 with the whole instrument was 0.92, 0.94, 0.91, 0.95, 0.93, and 0.91 respectively. In addition, a chi-square of 14.31 (df = 7, P = 0.001) showed good fitness. The GFI in the current study was 0.96, which showed the good fitting of the uni-dimensional model of the PTES construct. Further indices tested in this model were RMSEA = 0.01, CFI = 0.96, NFI = 0.95, and TLI = 0.97. All of the tested indices demonstrated that the extracted model was a good fitting (Fig. 2).

### Reliability (internal consistency and stability)

#### Internal consistency

For evaluation of internal consistency with the Cronbach’s alpha across the 36 item-instrument was 0.96, which indicates the appropriate internal consistency of this questionnaire (Table 3).

**Table 3:** Cronbach’s alpha of subscales and the entire medication safety competence scale (MSCS).

Factors	Subscale	Items	Cronbach’s alpha
1	Patient-centered medication management	9	0.96
2	Improvement of safety problems	8	0.92
3	Management of effecting factors	6	0.89
4	Safety risk management	6	0.96
5	Multidisciplinary collaboration	4	0.88
6	Responsibility in the nursing profession	3	0.95
Entire questionnaire		36	0.96

#### Stability

The mean (standard deviation) for medication safety competence scale was 139.31(18.39), also the intra-class correlation coefficient across the 36 item-instrument was 0.90, which indicates the appropriate internal consistency of this questionnaire (Table 4).

**Table 4:** Mean (standard deviation) and intraclass correlation coefficient (ICC) values for the domains of the medication safety competence scale (MSCS).

Factor	Dimensions	Mean ± SD	ICC	Confidence interval	P-value
1	Patient-centered medication management	35.17 (6.44)	0.89	0.76–0.89	p < 0.05
2	Improvement of safety problems	28.54 (6.11)	0.98	0.877–0.99	p < 0.05
3	Management of effecting factors	20.35 (5.28)	0.93	0.84–0.94	p < 0.05
4	Safety risk management	19.11 (5.01)	0.97	0.88–0.99	p < 0.05
5	Multidisciplinary collaboration	18.08 (6.13	0.87	0.79–0.95	p < 0.05
6	Responsibility in the nursing profession	18.06 (5.43)	0.92	0.87–0.98	p < 0.05
Safety competence scale (total)		139.31 (18.39)	0.90	0.86–0.92	p < 0.05

#### Determination of ease of use of the questionnaire

To determine the ease of use of the questionnaire, the researchers used the average length of time it took to complete the instrument, which was calculated to be 9 min (in a range of 5 to 13 min). The mean was calculated based on how much time the participants needed to complete the questionnaire. The non-response rate was between 0 and less than 5 percent. The final version of the MSCS is a self-report questionnaire based on a five-point Likert scale scoring system (1–5). MSCS consists of 36 items classified into six dimensions. The total score range is from 36 to 180. A score between 36 and 75 indicates poor medication safety competence, 76 and 130 indicates moderate medication safety competence and 131 and 180 indicates favorable medication safety competence.

## Discussion

The present study was conducted to translate and evaluate a Persian version of medication safety competence scale for clinical nurses in the south of Iran. The findings of the study showed that, like the original version of the scale, the Persian version of the medication safety competence scale for nurses was adequately valid and reliable and none of the 36 items of the questionnaire were omitted. Evaluation of the face validity of the scale showed that all the 36 items had an impact score of above 1.5 and, therefore, none of them were omitted. In addition, evaluation of the content validity of the scale showed that the CVR of each item ranged between 0.76 and 1, which is a satisfactory value^29^. The, I-CVI of the scale was found to be between 0.80 and 1, and S-CVI was a satisfactory 0.94^30^. Similarly, in the study of Yang et al., all the 36 items of the Chinese version of the medication safety competence scale for nurses were found to possess satisfactory reliability and validity and none of the items were omitted. Yang reported the I-CVI of the scale to be between 0.85 and 1 and the index of S-CVI to be 0.95^31^. Unlike the present study, Yang’s study did not evaluate the face validity of the scale, which is one of the strengths of the present study.

In the present study, exploratory factor analysis showed that 6 factors explained 84.94% of the variance and the factor loading of the items was between 0.72 and 0.87, which is at a satisfactory level. Likewise, the results of the study of Yang et al. showed that, after exploratory factor analysis, the six domains of the Chinese version of the medication safety competence scale accounted for 71.48% of the variance and the factor loading of the items was between 0.57 and 0.88, which is considered satisfactory^31^. As for the original version of the scale, Park and Seomun, the developers of the instrument, reported that the results of exploratory factor analysis showed that the 6 domains of the medication safety competence scale explained 63.2% of the variance^14^.

The confirmatory factor analysis, the average variance extracted values in present study were 0.72 to 0.87, and the model fitting indexes were all in the acceptable range. Similarly, in the study of Yang et al., states the average variance extracted values were 0.55 to 0.70, in the confirmatory factor analysis, and the model fitting indexes were all in the acceptable range^31^. Also, the confirmatory factor analysis, the average variance extracted values in present study were 0.62 to 0.76, and that the hypothesized factor structure was a good fit^32^.

The results of the present study showed that the Persian version of the medication safety competence scale for nurses possesses a satisfactory degree of reliability: The Cronbach’s alpha of the six domains of the scale was found to range between 0.88 and 0.96. The Cronbach's alpha of the whole instrument was found to be 0.96. Moreover, the intra-class correlation coefficient (ICC) of the entire scale was a satisfactory 0.90^33^. Similarly, the study of Yang et al. showed that the Chinese version of the medication safety competence scale is adequately reliable: they reported that the Cronbach’s alpha coefficients of the six domains of the scale were between 0.84 and 0.94 and the intra-class correlation coefficient (ICC), of the whole scale was 0.70, which is satisfactory^31^. In their evaluation of the reliability of the original version of the scale, Park and Seomun reported the Cronbach’s alpha coefficients of the six domains of the scale to be between 0.77and 0.96 and the intra-class correlation coefficient (ICC) of the whole scale to be a satisfactory 0.78^14^.

### Limitations

The target population of the present study was nurses and nursing students were not included. Therefore, it is suggested that future research incorporate the views of nursing students too. The present study did not address the contributory factors in medication safety (it was not among the objectives of the study). It is suggested that future research address these factors. In addition, given the cultural differences between different countries, it is recommended that this scale be translated and evaluated in other countries too. As the medication safety competence scale had been translated and evaluated only in China, the researchers in the present study could compare their findings to the results of Yang’s study only, which constitutes another limitation of the present study**.**

### Strengths

In the present study, the psychometric properties of the instrument were evaluated according to COSMIN criteria. Also, the researchers used a large sample of nurses from different hospital departments.

## Conclusion

The Iranian version of the medication safety competence scale is sufficiently reliable and valid. Therefore, nurse managers can use this instrument to measure medication safety competence in nurses. It is also recommended that nursing instructors consider evaluating nursing students’ medication safety competence in their educational programs. The findings of the study can be used as a useful resource for development of a competence-based education program that promotes medication safety.

## Data Availability

The data that support the findings of this study are available from the corresponding author upon reasonable request.

## Abbreviations

MSCS: Medication safety competence scale
COSMIN: Consensus-based standards for the selection of health measurement instruments
ICU: Intensive care unit
CCU: Coronary care unit
CVR: Content validity ratio
S-CVI /Ave: Scale-level content validity index/average
KMO: Kaiser–Meyer–Olkin Index
ICC: Intraclass correlation coefficient
